# Supplementary material for: Histone Methyltransferase SsDim5 Regulates Fungal Virulence through H3K9 Trimethylation in Sclerotinia sclerotiorum
Source: J Fungi (Basel). 2024 Apr 6;10(4):271. doi: 10.3390/jof10040271 (PMC11051235; doi:10.3390/jof10040271)
Supplement: Supplementary file 1 [file jof-10-00271-s001.zip › jof-2930492-supplementary.pdf]

Supplementary Figures

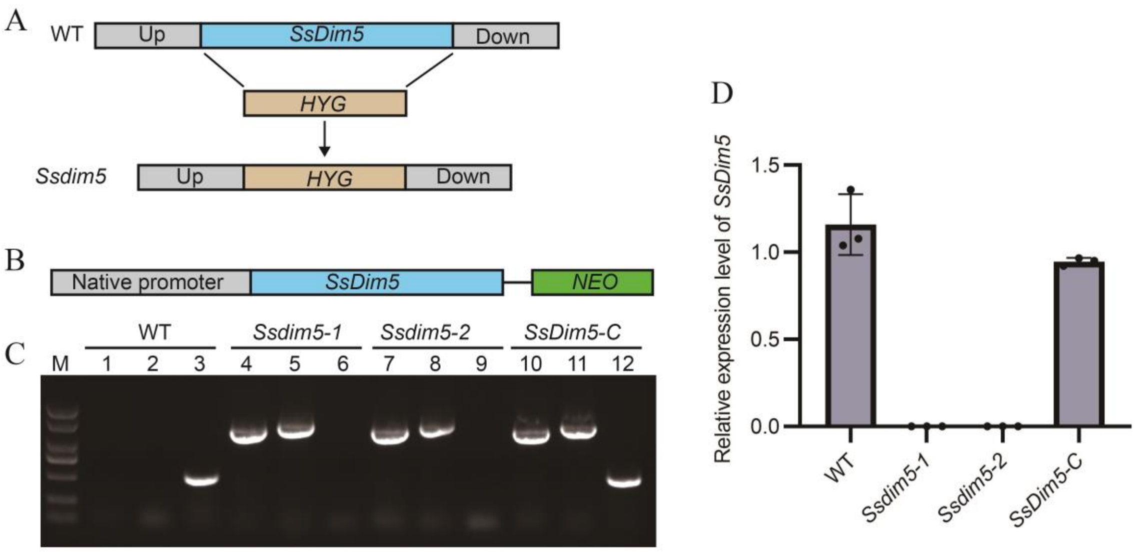

Supplementary Figure 1. Identification of *SsDim5* knockout mutants and genetically complemented strains. (A) Schematic diagram of the *SsDim5* knockout method. (B) Schematic diagram of the *SsDim5* complementation method. (C) PCR verification. Primers Dim5-up-check-F/hyg-check-R amplify the upstream flanking sequence of the *SsDim5* gene and part of the TrpC promoter fusion fragment (lanes 1, 4, 7 and 10), and primer hyg-check-F/ Dim5-down-check-R amplifies The downstream flanking sequence of the *SsDim5* gene and a part of the TrpC-terminator fusion fragment (lanes 2, 5, 8 and 11), and the primer Dim5-F/Dim5-R amplified the *SsDim5* genomic fragment (lanes 3, 6, 9 and 12). (D) Quantitative real-time PCR detection of *SsDim5* transcripts in WT, *Ssdim5-1*/*Ssdim5-2*, and *SsDim5-C*. Utilizing *SsTubulin1* as the reference gene, average values and standard deviations were computed based on data from three independent biological replicates.

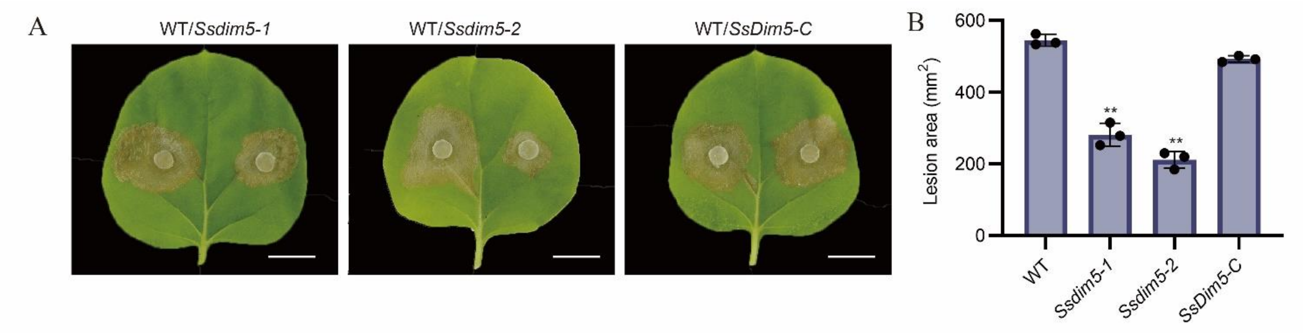

Supplementary Figure 2. Pathogenicity assays of individual strains in *N. benthamiana* leaves. (A) Disease phenotype of WT, *Ssdim5-1*, *Ssdim5-2* and *SsDim5-C* of *N. benthamiana* leaves. Bar = 1cm. Photographs were taken at 24 hpi. One representative biological replicate was shown. (B) Statistical analysis of the lesion area in panels. WT, *Ssdim5-1*, *Ssdim5-2* and *SsDim5-C*. Average values and standard deviations were computed based on data from three independent biological replicates. Differences were evaluated using the one-way ANOVA test. * denotes $P < 0.05$, ** denotes $P < 0.01$.

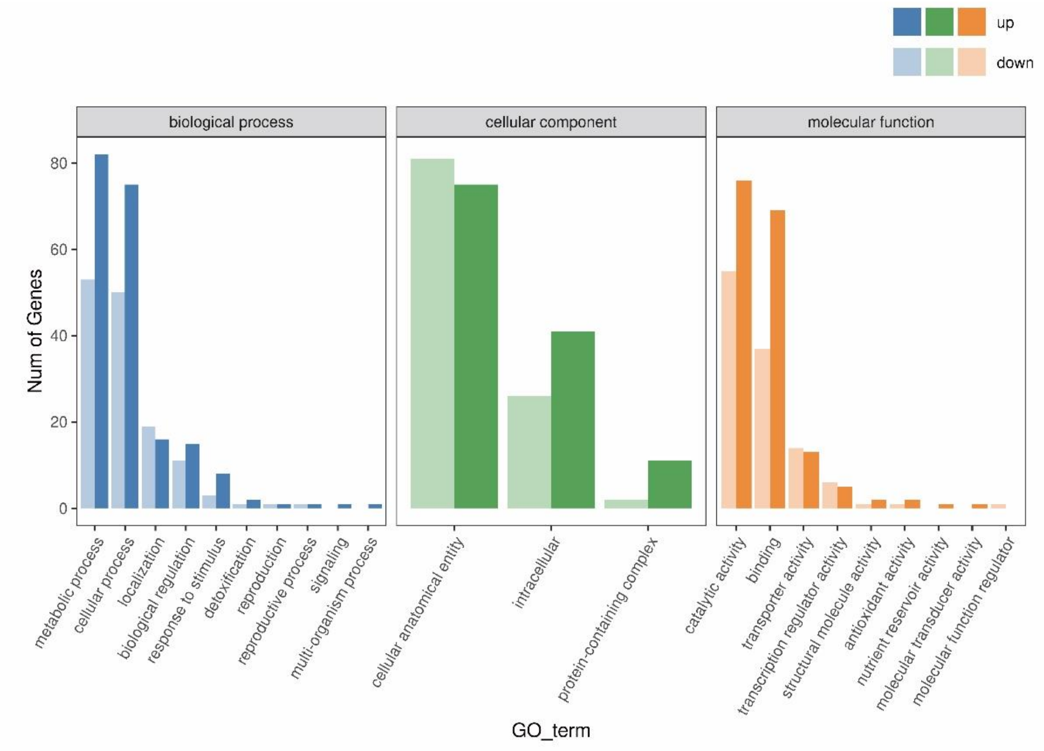

Supplementary Figure 3. GO enrichment analysis of differentially expressed genes in *SsDim5* mutants.

Supplementary Tables

Table S1: Primers used in the experiment

Dim5-up-F	TCTTCTTCGCACCTGCCTTC
Dim5-up-R	AATGGGTTGAATAAGTATCCAG
Dim5-down-F	TACGCGCAAGGATGATCTAT
Dim5-down-R	CCAACTTTCTAGCGAGTCAA
Dim-F	TGGTGCCAAATTCGCAGCTT
Dim-R	TATTAGGTTCGCAGGAGTGA
Dim5-up-check-F	TACGCTGCGCATCCGACCCC
Dim5-up-check-R	TGTGGCGTCGTCTTTGTTGA
HYG-F	AGAAGATGATATTGAAGGAGCAC
HY-R	GCATCATCGAAATTGCCGTCAACC
YG-F	TCTCGGAGGGCGAAGAATCTCGTGC
HYG-R	AAAGAAGGATTACCTCTAAACAAGTGT
HY-CHECK-R	TTCATTGTTGACCTCCACTA
YG-CHECK-R	TACAGGACACACATTCATCG
Dim5-Com-F	TCTCGTCACAAGCGCAAGCT
Dim5-Com-R	TCTCAATTCTCAGCTTTCGA
SsTublin1-RT-F	CGATGAAGCTCAATCCAAACGA
SsTublin1-RT-R	CAGAGTCGAGCACAATACCG
SsDim5-RT-F	TCTAACTCTCCCTGGTGATTT
SsDim5-RT-R	TCCTTCACTATTACCACCTG

SS1G_02251-RT-F	TTACGATTATTACCACGA
SS1G_02251-RT-R	GAACATCACTTTCCTTTG
SS1G_07229-RT-F	GTCAGTCTTTTCGTCGTT
SS1G_07229-RT-R	TCATTGGCACCACTATTT
SS1G_13355-RT-F	gcattgggaaaggatagtc
SS1G-13355-RT-R	catcgtcgtgattgtggtc
SS1G_13358-RT-F	atctcgccatacttcttc
SS1G_13358-RT-R	agtcttctccccaaaatc
SS1G_13636-RT-F	tacgaaataccgaggaaag
SS1G_13636-RT-R	gtaggactatcaggcaccc
SS1G_13850-RT-F	TCAAAGGTCCTCCATCCC
SS1G_13850-RT-R	TACGAAAGCCATTCTGCC
